# A High-Speed Low-Cost VLSI System Capable of On-Chip Online Learning for Dynamic Vision Sensor Data Classification

**DOI:** 10.3390/s20174715

**Published:** 2020-08-21

**Authors:** Wei He, Jinguo Huang, Tengxiao Wang, Yingcheng Lin, Junxian He, Xichuan Zhou, Ping Li, Ying Wang, Nanjian Wu, Cong Shi

**Affiliations:** 1 School of Microelectronics and Communication Engineering, Chongqing University, Chongqing 400044, China; hewei007@cqu.edu.cn (W.H.); jg_huang@cqu.edu.cn (J.H.); tengxiao.wang@cqu.edu.cn (T.W.); linyc@cqu.edu.cn (Y.L.); junxian_he@cqu.edu.cn (J.H.); zxc@cqu.edu.cn (X.Z.); lipingstu@cqu.edu.cn (P.L.); 2 Key Laboratory of Dependable Service Computing in Cyber Physical Society (Chongqing University), Ministry of Education, Chongqing 400044, China; 3 State Key Laboratory of Computer Architecture, Institute of Computing Technology, Chinese Academy of Sciences, Beijing 100190, China; wangying2009@ict.ac.cn; 4 State Key Laboratory of Superlattices and Microstructures, Institute of Semiconductors, Chinese Academy of Sciences, Beijing 100083, China; nanjian@red.semi.ac.cn

**Keywords:** address-event representation (AER), Random Ferns, object classification, neuromorphic hardware, online learning, on-chip learning

## Abstract

This paper proposes a high-speed low-cost VLSI system capable of on-chip online learning for classifying address-event representation (AER) streams from dynamic vision sensor (DVS) retina chips. The proposed system executes a lightweight statistic algorithm based on simple binary features extracted from AER streams and a Random Ferns classifier to classify these features. The proposed system’s characteristics of multi-level pipelines and parallel processing circuits achieves a high throughput up to 1 spike event per clock cycle for AER data processing. Thanks to the nature of the lightweight algorithm, our hardware system is realized in a low-cost memory-centric paradigm. In addition, the system is capable of on-chip online learning to flexibly adapt to different in-situ application scenarios. The extra overheads for on-chip learning in terms of time and resource consumption are quite low, as the training procedure of the Random Ferns is quite simple, requiring few auxiliary learning circuits. An FPGA prototype of the proposed VLSI system was implemented with 9.5~96.7% memory consumption and <11% computational and logic resources on a Xilinx Zynq-7045 chip platform. It was running at a clock frequency of 100 MHz and achieved a peak processing throughput up to 100 Meps (Mega events per second), with an estimated power consumption of 690 mW leading to a high energy efficiency of 145 Meps/W or 145 event/μJ. We tested the prototype system on MNIST-DVS, Poker-DVS, and Posture-DVS datasets, and obtained classification accuracies of 77.9%, 99.4% and 99.3%, respectively. Compared to prior works, our VLSI system achieves higher processing speeds, higher computing efficiency, comparable accuracy, and lower resource costs.

## 1. Introduction

Visual data analysis is a hot topic in scientific research, and is widely applied in many fields, such as smartphones [1], automatic driving [2], surveillance cameras [3], and smart healthcare [4]. Most of these systems collect vision data using conventional cameras. However, this type of sensor suffers from some drawbacks. First, traditional visual sensors acquire visual information as a series of snapshot images composed of a large number of pixels at a fixed rate, even though most of the sense does not change, causing a huge data redundancy and energy waste. Second, the computer vision system cannot analyze and process the image data from a traditional camera until one image frame has been sent out, which cannot be tolerated in real-time applications requiring low latency [5]. To find a way to represent vision information more efficiently, researchers turn to bio-inspired vision sensors, in which information is coded and communicated by spikes [6,7,8,9,10]. A bio-inspired frame-free camera was proposed in [11], namely, a dynamic vision sensor (DVS). A DVS camera mimics the way human retina works and generates a stream of pixel-level spike events, called address-event-representation (AER), to represent the temporal change in illumination [10]. These spike events are sent out from DVS in the format of (*x*, *y*) indicating the position in the pixel array. The DVS will be inactive and does not output AER spikes when there is no moving object, and light intensity does not change in scenes. Therefore, visual systems based on DVS cameras with spatiotemporally sparse spike trains are more energy efficient than those equipped with traditional frame-based image sensors. 

However, designing proper algorithms and hardware systems to efficiently process the AER data is still challenging, as many real-time embedded systems prefer low processing latency, requiring the AER data generated by DVS cameras to be consumed as soon as possible. To meet this goal, researchers have designed many digital programmable neuromorphic processors running spiking neural network (SNN) algorithms [12,13,14,15,16] to process AER spike streams. These digital processors include SpiNNaker [17,18], TrueNorth [19,20], Loihi [21], Darwin [22], ODIN [23], and spiking Convnets chips for deep spiking convolutional neural networks (SCNN) [24,25,26,27], to name a few. Nevertheless, these systems are designed to be functionally generic to implement versatile SNNs of different spiking neuron models, network topologies, and learning algorithms. Many of them suffer from high computing complexity and large resource consumptions, and are not optimal for embedded high-speed application scenarios. In particular, the TrueNorth, SpiNNker, and Loihi chips are primarily designed to provide a large-scale platform for human brain behavior emulation in the neuroscience research field, rather than for solving computer vision or machine learning problems. These chips are more generally functional and consume a significant amount of space even using advanced nanometer silicon technologies (i.e., SpiNNaker: 102 mm^2^/180 nm, TrueNorth: 430 mm^2^/65 nm, Loihi: 60 mm^2^/14 nm). These cost-expensive chips are not attractive to many embedded vision systems under tight cost budgets. This limitation motivates us to design fast and cost-efficient AER processing hardware systems based on algorithms with low computational complexity. 

Recently, some researchers have resorted to lightweight methods based on conventional shallow statistical learning rather than spiking neural networks to process AER streams [28,29]. These methods greatly reduce computing complexity and maintain high classification accuracy. For instance, Bag of Events (BoE) features are proposed to represent the AER stream as joint probability distribution of the events [28]. However, it still uses complicated classifiers such as the Support Vector Machine (SVM) [28] or Random Forests (RF) [29] for feature classification. These classifiers are more difficult to train with a few hardware circuits on the chip (on-chip learning) than on expensive general-purpose computers, or trained by feeding individual training samples one after another in-situ (online learning) when the training samples are impossible to collect beforehand. Therefore, the BoE algorithm is limited in many embedded or mobile applications where on-chip online learning is compulsory. 

To overcome the above problems, a lightweight AER classification algorithm is proposed in [30] based on extremely computationally simple binary features and the Random Ferns classifier. According to software simulations, the algorithm can be executed 2–3 orders of magnitude faster than prior works and achieves comparable classification accuracy [30]. In particular, the Random Ferns classifier inherently supports fast and simple online learning involving merely a few counting operations [31], which provides a good opportunity for on-chip learning with simple hardware circuits. 

Therefore, in this paper, we propose a high-speed low-cost VLSI hardware system dedicated to AER object classification tasks with fast on-chip online learning capabilities [32], based on such lightweight statistical algorithms utilizing the Random Ferns classifier [30]. The proposed system mainly consists of 3 modules: a motion detector, a bank of binary feature extractors, and a Random Ferns engine. The system architecture adopts multi-level pipelines and parallel processing units to realize high-speed performance and maximize AER data throughput. Moreover, our hardware system is realized in a cost-effective memory-centric paradigm, with which an FPGA prototype was implemented with 9.5~96.7% block memory consumption and <11% computational and logic resource consumption on a Xilinx Zynq-7045 chip platform. In addition, the proposed system is capable of on-chip online learning to flexibly adapt to different in-situ application scenarios, such as tiny embedded and mobile devices. The extra overheads for on-chip learning regarding time and resource consumption are quite low, as the learning procedure of Random Ferns is very simple and requires few auxiliary learning circuits. 

The main contributions of our work are as follows: (1) We propose a high-speed low-cost VLSI hardware system dedicate for AER object classification based on a lightweight statistical algorithm. The system reaches a peak throughput up to 100 Meps (measured with a virtual DVS sensor) and a high energy efficiency of 145 Meps/W, while achieving comparable classification accuracies. (2) We optimized and improved the hardware friendliness of the used statistical algorithm originally proposed in [30], so that it can be carried out on the hardware without expensive multipliers and dividers. (3) We propose multi-level pipeline schemes with massively parallel processing circuits to maximize the system processing speed. (4) We have designed on-chip online learning circuits to make our AER processing system cost-effective as well as adaptive to embedded and mobile devices. (5) An FPGA prototype was implemented and elaborate experiments on different DVS datasets were conducted and analyzed, which can be connected to a real DVS retina for building a complete AER system in future work.

This work has partly been released in [32,33]. In [33], a premature version of our work without on-chip online capability was reported, where the classifier parameters of the Random Ferns engine had to be learned offline on a general-purpose computer. Later on, our completed system incorporating on-chip online learning was reported in [32]. However, both Ref. [33] and [32] are brief conference reports, with neither sufficient design details nor elaborate experimental results on more DVS datasets other than MNIST-DVS. In contrast, this paper covers all of the system architecture and circuit design details with sufficient experimental measurements on more DVS datasets: Poker-DVS and Posture-DVS. Comparison to more recent state-of-the-arts and discussions are also provided. Moreover, the VLSI design was slightly optimized and re-organized in a better hierarchy in this paper.

The rest of this paper proceeds as follows. Section 2 reviews the lightweight AER classification algorithm [30] deployed on our VLSI hardware system. The details of the proposed VLSI system architecture and circuits are revealed in Section 3. In Section 4, we show the experimental results with further discussion. Finally, our conclusions are made in Section 5. 

## 2. Algorithm Review

Our hardware system for AER object classification is based on the lightweight statistical algorithm proposed in [30]. For integrity of this paper, we will briefly review this algorithm. Some algorithm optimizations for ease of hardware implementation are provided.

### 2.1. Algorithm Flow

The algorithm flow for AER object classification used in this work is as follows [30]. To avoid taking on classification upon the arrival of every spike event, an AER stream segmentation scheme is employed [28]: every *α* successive events in the AER stream are grouped as a segment, and feature extraction and classification are carried out on each such segment instance. The value of *α* is manually set according to different DVS datasets or application requirements. Intuitively, it should be larger in a slow-moving scenario to collect more AER spikes for a meaningful motion segment, and vice versa. 

To extract the features of AER segments, *M* groups × *S* pairs/group of square patches are randomly selected within the pixel array plane. The sizes (denoted as *D* × *D*) and locations of these patches are randomly and independently selected at the very beginning, and then kept unchanged throughout. For each patch pair, one is regarded as positive and the other negative. For an AER segment, if the number of spike events with their addresses (*x*, *y*) falling into the negative patch exceeds that falling into the positive patch, a binary feature of value 1 is asserted for the AER segment. Otherwise, a binary feature of value 0 is asserted. The *S* binary features within one group of the patch pairs are concatenated as an *S*-bit feature. The *S*-bit features from all the *M* groups of patch pairs are fed to the Random Ferns classifier for training or inference. We can see that the feature extraction procedure is rather simple, as it merely involves a few counting and comparison operations. 

The Random Ferns classifier is responsible for feature classification. It has *M* ferns, holding a one-to-one correspondence to the *M* patch groups. We define *F_m_* as the evaluated numeric value of the *S*-bit feature fed to the *m*-th fern. Take *S* = 5 for example, if an extracted 5-bit features fed to the *m*-th fern are (0, 1, 0, 1, 0), then *F_m_* = (01010)_2_ = 10. There will be 2*^S^* different possible feature values for each fern. We chose the Bayesian way to combine these ferns as our classifier [30]. Assume *C* is the number of the object classes and *c* ∈ {0, 1, …, *C* − 1} as the class label. During training, we only needed to learn two sets of tensors from labeled AER segments: (1) *N*(*m*, *F_m_*, *c*), a 3-D tensor storing the number of training segments with label *c* and their *S*-bit feature corresponding to the *m*-th fern evaluated as *F_m_*, with *m* running from 0 to *M* − 1, *F_m_* from 0 to 2*^S^* − 1, and *c* from 0 to *C* − 1; and (2) *N_c_*, a 1-D tensor (i.e., a vector) storing the total number of training segments with label *c*, with *c* running from 0 to *C* − 1. The learning process is rather simple and can be executed online; when an AER segment labeled as *c* arrives, we extract its features *F_m_*, and increase corresponding *N_c_* and *N*(*m*, *F_m_*, *c*) entries in each fern by 1. During inference, when an unlabeled AER segment arrives, we extracted its features *F_m_* and determine its category c* by calculating the maximum posterior probability across all classes [30]:

(1) $$c^{*}=\underset{c}{\arg\max}\prod_{m=0}^{M-1}P(F_{m}|c)$$

where the class-conditional probability is expressed as:

(2) $$P(F_{m}|c)=\frac{N(m,F_{m},c)+N_{r}}{N_{c}+2^{S}N_{r}}$$

with *N_r_* = 1 as a regularization prior. Refer to [30] for more algorithm details.

### 2.2. Algorithm Optimization 

To make this algorithm more hardware-friendly, we carried out some optimizations on it. As described in Equations (1) and (2), the inference procedure in Random Ferns contains multiplication and division operations, which require many expensive computing resources and result in high system latency. To solve this problem, we converted Equation (1) into (3) via a logarithmic transform [32]:

(3) $$\begin{array}{ll} c^{*} & =\underset{c}{\arg\max}\prod_{m=0}^{M-1}P(F_{m}|c)\underset{c}{=\arg\max}\sum_{m=0}^{M-1}\log P(F_{m}|c)\\ & =\underset{c}{\arg\max}\sum_{m=0}^{M-1}\log\frac{N(m,F_{m},c)+N_{r}}{N_{c}+2^{s}N_{r}}\\ & =\underset{c}{\arg\max}(\sum_{m=0}^{M-1}\log(N(m,F_{m},c)+N_{r})-M\log(N_{c}+2^{s}N_{r})) \end{array}$$

The logarithmic function above can be implemented at high speeds by a small lookup table (LUT) on-chip memory. Expensive hardware multipliers and dividers are mostly removed. Since on-chip memory can achieve very high density under nanoscale semiconductor technology, its cost is much lower than that of the computational circuits. Therefore, the overall hardware system cost is reduced. 

## 3. VLSI Hardware System

### 3.1. System Architecture

**Figure 1:**
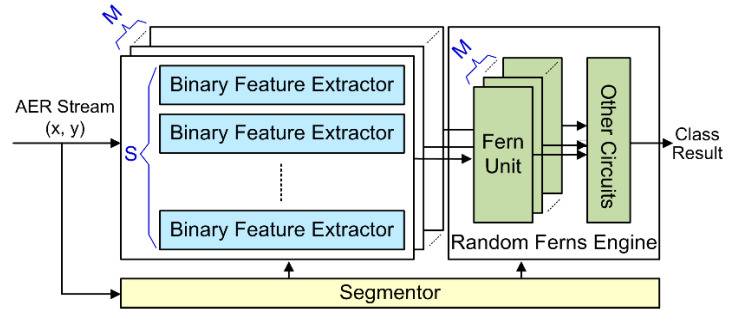
Hardware architecture of the proposed VLSI system.

**Figure 2:**
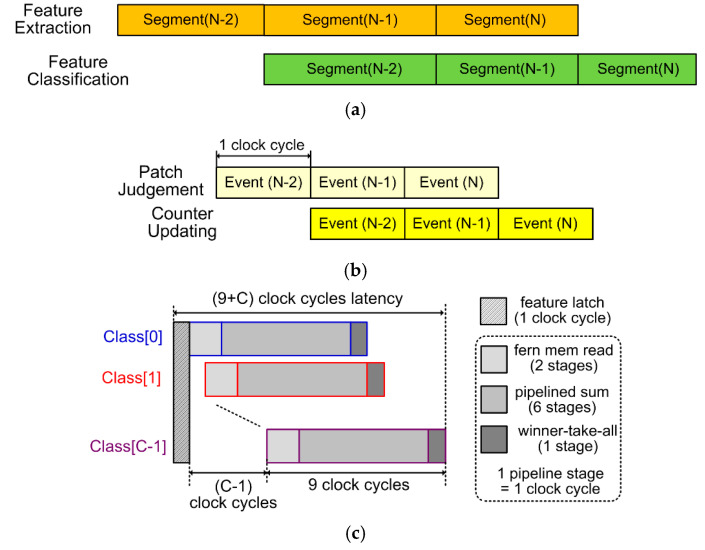
Multi-level pipelines. (**a**) The course-grained segment-level pipeline. (**b**) The fine-grained event-level pipeline in the feature extractors. (**c**) The fine-grained feature-level pipeline in the Random Ferns engine.

The overall hardware architecture of the proposed VLSI system is shown in Figure 1. It consists of a segmentor block, a bank of *M* × *S* binary feature extractors, and a Random Ferns classifier engine with on-chip online learning functionality. The segmentor block monitors the number of incoming AER spike events from a DVS camera. Once the number of events counts to *α* signaling the end of an AER segment, the segmentor latches the AER segment features extracted by the feature extractors, and triggers the Random Ferns engine to start training or inference on these latched features. After that, the segmentor and feature extractors are reset to handle the next AER segment. The proposed system architecture has the following characteristics: 

*(1) Multi-level pipelines and parallel processing:* To maximizing data throughput, a multi-level pipeline scheme is utilized in our system. In the course-grained 2-stage segment-level pipeline, as depicted in Figure 2a, the Random Ferns engine carrying out training or inference on the features of the previous AER segment is pipelined with the feature extractors processing and extracting features from current AER segment. As will subsequently be exhibited, the Random Ferns engine only takes up 3 or (9 + *C*) clock cycles to process features of one AER segment for training or classification inference, respectively, which is ignorable compared to the duration of an AER segment occupying hundreds to thousands of clock cycles. Thus, time consumption for Random Ferns are completely hidden behind feature extraction, and the system throughput is significantly improved. Note that the number of clock cycles for each pipeline stage is always bottlenecked by the feature extractors, and varies depending on the time duration of each AER segment. To support the segment-level pipeline, fine-grained pipelines, i.e., the event-level pipeline inside the feature extractors and the class-level pipelines inside the Random Ferns engine as shown in Figure 2b,c, respectively, are employed in our system. The circuit implementation details of the two pipelines will be given in Section 3.2. Here we just briefly illustrate their timings. The event-level pipeline in feature extractors contains two stages, each occupying only one clock cycle. In other words, this pipeline has a throughput as high as 1 event per clock cycle, with a pipeline latency of 2 clock cycles. The class-level pipeline has 9 stages, each also occupying one clock cycle. It enables the Random Ferns engine to finish feature classification within a latency of (9 + *C*) clock cycles, by pipelining the fern tensor and LUT memory reads, logarithmic class-conditional probability summation, and winner-take-all (WTA) operations for each class candidate, as required by Equation (3). Note that in Figure 2c the feature latching step consumes 1 clock cycle. This step is out of the class-level pipeline, but still contributes to the overall processing latency for AER segment feature classification in the Random Ferns engine. 

To boost the processing performance and satisfy those tight pipeline timing constraints, the proposed system architecture employs parallel processing circuits, including the massively parallel binary feature extractors, as well as parallel fern units, as shown in Figure 1. These pipeline schemes and parallel units together enable the feature extractors to immediately consume an incoming spike event at a maximum throughput of 1 event per clock cycle, and the Random Ferns to process the extracted features of one AER segment in no more than (9 + *C*) clock cycles. This eventually leads to an extremely high overall system throughput of 1 AER spike event per clock cycle, equivalent to 100 Meps under a moderate 100 MHz system clock frequency. 

*(2) Pseudo-simultaneity:* The concept of the *pseudo-simultaneity* property in AER systems was earlier proposed in [24,34]. Their AER convolutional processing circuits can consume and process one event on-the-fly in as short as 0.175 μs. Therefore, once the DVS provides sufficient events (e.g., one AER segment) representing the reality sense, the AER processing result immediately appears at a latency far less than 1 ms, called *pseudo-simultaneity*. This property is unique for AER systems and hard to realize in conventional frame-based visual systems where the latency is as long as a frame interval often around 30 ms. In our system, such *pseudo-simultaneity* latency is merely 2 + (9 + *C*) = 11 + *C* clock cycles, the event-level pipeline latency in feature extractors plus the time to classify features of one AER segment in the Random Ferns engine, as shown in Figure 2. If *C* = 10 object classes which is a typical case for AER-based applications, and if the clock cycle is 10 ns, which is easy to reach on nanotechnology VLSI chips, as demonstrated by our experiments later, the overall latency is only 0.21 μs, thus the pseudo-simultaneity property is consolidated in our system. 

*(3) Cost-effective memory-centric paradigm*: The proposed VLSI hardware system design is dedicate to carrying out the lightweight AER classification algorithm in [30]. The computing operations in this algorithm are quite few and simple. But it requires moderately sufficient memory space to store the learned Random Ferns tensors *N*(*m*, *F_m_*, *c*) and *N_c_*, as introduced in Section 2.1, as well as to implement LUTs for the logarithm computations required by Equation (3). Therefore, the hardware system is naturally memory-centric, consuming a moderate amount of memory and very few affiliated computational units. For instance, Section 4 shows that an FPGA prototype of the proposed VLSI system consumes 9.5~96.7% Block RAMs and <11% other logic resources available on a medium-cost Xilinx Zynq-7045 platform. Since the memory cells have much higher density under nanometer silicon technology, the memory resources are much cheaper than the computational and logic resources. As a result, the proposed system architecture with memory-centric paradigm is cost effective. 

*(4) On-chip online learning capability:* The proposed VLSI system supports online learning by on-chip circuits. As stated earlier in Section 1, online learning means that the Random Ferns classifier is trained and updated in-situ upon the unlabeled training AER segments one by one, and on-chip learning eliminates the need to incorporate an expensive general-purpose computer for off-chip training. A minimized number of extra circuits are needed for on-chip learning, the Random Ferns training procedure involves only a few simple counting operations to obtain the required *N*(*m*, *F_m_*, *c*) and *N_c_* tensors, as described in Section 2.1. Therefore, the on-chip online learning capability facilitates our system to be widely and adaptively applied in versatile embedded or mobile scenarios with in-situ learning requirements and a tight cost budget. The details of the Random Ferns circuits incorporating the learning functionality are exhibited in the next subsection. 

*(5) Scalability:* Finally, the proposed system architecture can be easily scaled up along the *M*, *S*, or *C* dimensions, to support more feature groups or ferns, more binary features in each group, or more AER object classes, respectively, to satisfy various applications with different classification accuracies and/or resource cost requirements. We describe how the FPGA prototype instances of our VLSI system with different amounts of resource consumption for 3 DVS datasets demonstrated such scalability in Section 4.1.

### 3.2. Circuit Design

**Figure 3:**
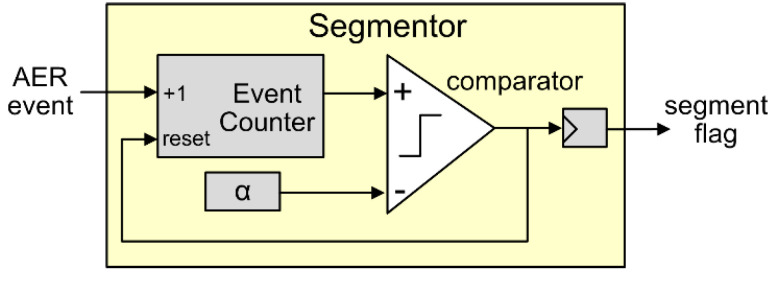
The circuit of the segmentor block.

*(1) Segmentor Block:* The circuit design of the segmentor block is shown in Figure 3. It consists of a 16-bit event counter, a comparator, and a 16-bit configurable parameter register storing *α*, the defined number of events in one AER segment, as introduced in Section 2.1. The event counter is initialized as 0 at the onset of one AER segment, and counts up incoming AER events (ignoring their addresses (*x*, *y*)). Once it reaches *α*, a segment flag signaling the end of the AER segment appears at the output. The flag is immediately sent out to latch extracted segment features into the Random Ferns engine and trigger Random Ferns training or inference, while simultaneously resetting the feature extractors and the segmentor itself to handle the next AER segment. To keep pace with the 2-stage event-level pipeline in feature extractors, the 1-bit flag is buffered by a flip-flop before it is outputted, as shown in Figure 3. 

**Figure 4:**
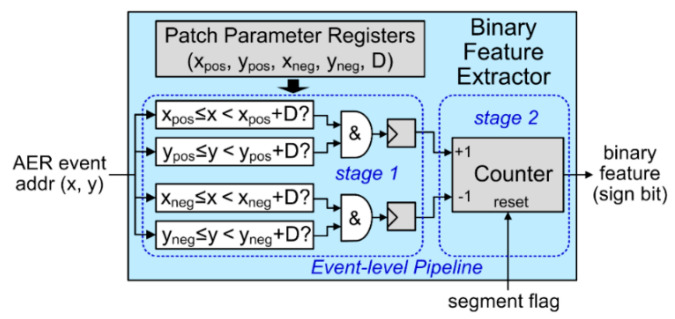
The circuit of the binary feature extractor.

*(2) Binary Feature Extractor: *Figure 4 shows the circuit of one binary feature extractor. When an AER event inputs, the event address is compared with the positions of a pair of positive and negative patches. The coordinate of the upper leftmost corner of the positive/negative patches are (*x*_pos/neg_, *y*_pos/neg_), and the patch sizes are *D* × *D*. These parameters are randomly selected as introduced in Section 2.1, and configured into the patch parameter registers before the system runs. An 8-bit bidirectional signed counter is employed and initialized to 0 at the onset of an AER segment. Note that the randomly selected pair of patches may overlap. When the address of an incoming event falls within the positive patch and not the negative one, the counter increases by 1. On the contrary, if the event address falls within the negative patch and not the positive one, the counter decreases by 1. Otherwise, the counter remains unchanged. When the AER segment is complete, the sign bit of the counter is the desired binary feature of the segment, as it indicates if the negative patch has seen more AER events than the positive patch. Compared to assigning two separate event counters for each of the positive and negative patches, using such a bidirectional counter saves as many as 8 bits × *M* × *S* flip-flop register resources for the massively parallel binary feature extractor array in Figure 1. For a typical case of *S* = 12 and *M* = 50, as in our later experiments, the total register reduction is 4800 bits. The 2-stage event-level pipeline schedule in Figure 2b is realized in the feature extractor circuit. The first stage judges if the event address falls into the patch regions in one clock cycle, and the second stage updates the bidirectional counter accordingly in another clock cycle. With this pipeline, an ultra-high event throughput of 1 event per clock cycle is achieved. 

**Figure 5:**
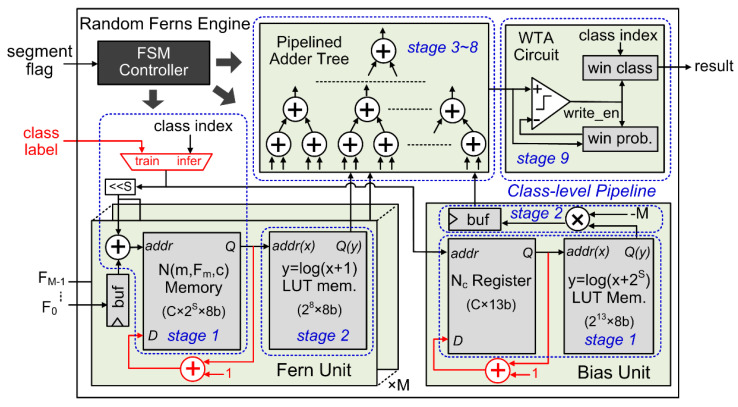
The circuit of the Random Ferns engine with fast on-chip online learning capability.

*(3) Random Ferns Engine:* The circuit details of the Random Ferns engine are depicted in Figure 5. The main components of the engine include: (1) an array of ferns units, each computing one log(*N*(*m*, *F_m_*, *c*) + *N_r_*) term in the last line of Equation (3); (2) a bias unit computing the −*M*log(*N_c_* + 2*^S^N_r_*) term; (3) a pipelined adder tree summing up all those terms; (4) a winner-take-all (WTA) circuit executing the *argmax*() function to obtain the classification result; and (5) a finite state machine (FSM) controller scheduling all operations in the engine. Block memories and registers are used to store the learned *N*(*m*, *F_m_*, *C*) and *N*_c_ tensors in the fern and bias units, respectively, while LUT memories are used to realize required logarithmic functions, with *N_r_* = 1 as said in Section 2.1. 

The feature classification procedure in the Random Ferns engine is accelerated by the class-level pipeline, as already illustrated in Figure 2c. This pipeline contains 9 stages, and each stage corresponds to one clock cycle. Immediately after the extracted AER segment features *F_m_* are latched into the buffer registers of the fern units, the Random Ferns engine evaluates Equation (3) on each class candidate and determine the final classification result in the pipeline, as follows. The FSM controller successively issues the candidate class indices *c* = 0, 1, …, *C* − 1 one at a clock cycle to the fern and bias units as well as to the WTA circuit at their respective proper timings. All the fern units and the bias unit run in parallel and they occupy the first 2 stages of the pipeline. In each fern unit, the first stage accesses the learned *N*(*m*, *F_m_*, *c*) memory element corresponding to feature *F_m_* and current class index *c*, and the other stage computes log(*N*(*m*, *F_m_*, *c*) + *N_r_*) as required in Equation (3) via a LUT memory, as shown in Figure 5. In the bias unit, as the few learned *N_c_* elements are stored in registers rather than a memory, they can be accessed without clock synchrony. Therefore, reading the *N_c_* element for current class index *c* and then computing the log(*N_c_* + 2*^S^N_r_*) term in Equation (3) via the bias unit’s LUT memory can be accomplished in one clock cycle, i.e., the first pipeline stage. The second stage multiplies the LUT output by a constant -*M*, as shown in Figure 5. Next, the outputs of these fern and bias units are accumulated in a 6-stage pipelined adder tree. We have validated that under a modern nanometer silicon technology, a 4-input 20-bit addition can be safely done within 10 ns. If this is the clock cycle period (as in our experiments), such a 6-stage adder tree can support up to *M* = 4^6^ − 1 (bias unit) = 4095 ferns, which are far too sufficient for any situations. If *M* is less than that, we can use less adders in the pipelined tree or less inputs of some adders to save unnecessary computing resources. The output stream of the adder tree is the logarithmic posterior class-conditional probability log*P*(*F_m_*|*c*) in Equation (3) of each candidate class index *c*. The last stage of the pipeline is assigned to the WTA circuit. In each clock cycle during the log*P*(*F_m_*|*c*) stream, if the current class index *c* has a higher log*P*(*F_m_*|*c*) than all previous ones, the current index *c* and its log*P*(*F_m_*|*c*) value are stored as the winner class and the winner probability, respectively, as Figure 5 illustrates. This way, the WTA circuit eventually picks out the class *c* with the largest log*P*(*F_m_*|*c*) as the classification result, in a pipelined manner with other circuits.

To realize the on-chip online learning capability of the Random Ferns classifier, only very few auxiliary hardware resources need to be consumed, those depicted in red in Figure 5. They are merely (*M* + 1) add-one calculators and a multiplexer, the costs of which are totally ignorable in the whole Random Ferns engine. The multiplexer chooses either the external class label of the training AER segment sample in the learning phase, or the class index stream internally generated by the FSM controller in the class inference phase. All entries of *N*(*m*, *F_m_*, *c*) and *N_c_* are initialized to 0 before any learning starts. Our Random Ferns engine equipped with these extra learning circuits consumes only 3 clock cycles to finish the learning on the features of a single training AER segment sample. The first cycle latches the extracted features *F_m_*. The second cycle reads corresponding *N*(*m*, *F_m_*, *c*) and *N_c_* memory entries in the parallel fern and bias units, according to *F_m_* and the class label *c* of the training AER segment. Finally, the last cycle adds the two items by 1 and then writes them back to their memories. 

## 4. Experimental Results

### 4.1. FPGA Prototype

**Figure 6:**
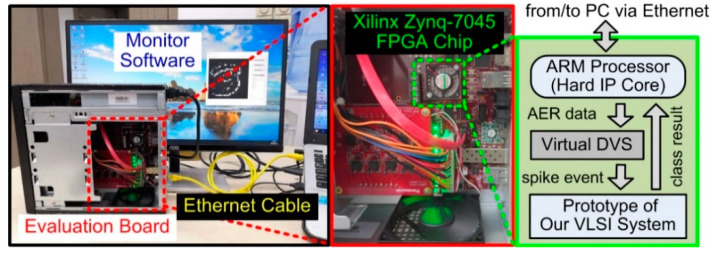
The FPGA prototype of the proposed VLSI system in the evaluation environment.

We implemented an FPGA prototype of the proposed VLSI system for AER data classification on a Xilinx Zynq-7045 chip mounted on a Mini-ITX evaluation board from the vendor AVNET [35], as shown in Figure 6. The FPGA chip contained our prototype, a virtual DVS camera, and a hard Intellectual Property (IP) core of an ARM processor. Our prototype was running under a clock frequency of 100 MHz and achieved a high AER event throughput up to 100 Meps, as mentioned in Section 3.1. The ARM core managed the overall prototype operations, and was connected to a host personal computer (PC) via a 1000 Mb/s Ethernet for communicating AER data and classification results between our prototype and the host PC. The virtual DVS sensor on the FPGA chip was essentially a 16 KB AER data buffer memory and it sent out stored AER event streams in a continuous manner, as a real DVS camera does. With such a virtualized sensor technique, the processing performance of the prototype in real application situations can be fairly measured, as if it was directly interfacing with a real sensor [36,37]. However, the virtual DVS and other components, such as the ARM core, the PC, and the Ethernet interface in Figure 6, were only used to build the laboratory evaluation environment. Their resource and time consumption overheads would no longer exist in a real application apparatus. A monitor software program was written on the PC for AER data preprocessing and visualizing, as well as displaying the classification result. 

**Figure 7:**
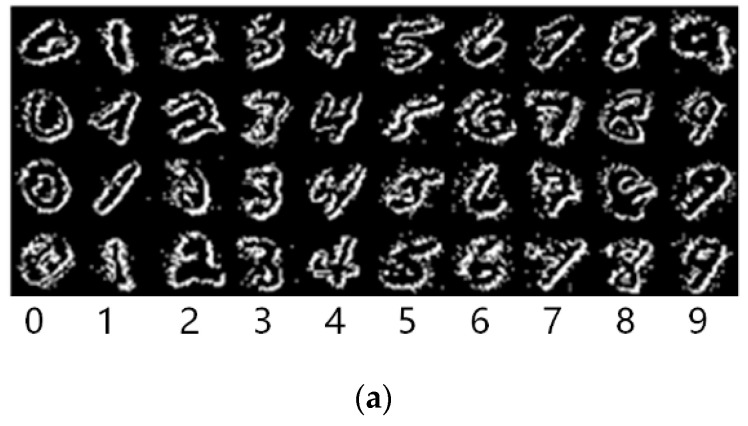

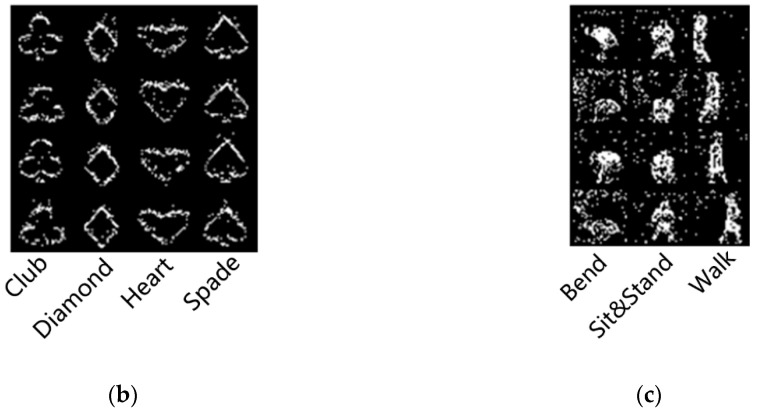
Some pseudo-images constructed from address-event representation (AER) segments of three dynamic vision sensor (DVS) datasets. (**a**) MNIST-DVS dataset with *C* = 10 classes. (**b**) Poker-DVS dataset with *C* = 4 classes. (**c**) Posture-DVS dataset with *C* = 3 classes.

We used three DVS datasets for evaluation: MINST-DVS with a 28 × 28 pixel resolution and 10 classes of handwritten digits from 0 to 9, Poker-DVS with a 32 × 32 resolution and 4 card classes of club, diamond, heart, spade, Posture-DVS with a 32 × 32 resolution and 3 human posture classes of bend, sit & stand, walk. The algorithm hyperparameters for processing these datasets are listed in Table 1. Figure 7 exhibits some *pseudo-images* reconstructed from the AER segments of the datasets [30]. These datasets had been generated by moving images in front of real DVS sensors and recorded with event timestamps [38]. However, to test the 100 Meps peak throughput of our prototype, we discarded all timestamps and implicitly regarded that one event was issued in every clock cycle in their original timestamp order. To validate the scalability, as mentioned in Section 3.1, the FPGA prototype was recompiled for each DVS dataset with different levels of resource and power consumption, as listed in Table 2. Note that the levels of consumption do not include those on the ARM core and the virtual DVS camera, because they were only used for evaluation and were not part of our prototype, as mentioned above. From the system and circuit designs in Section 3, we can deduce that: (1) the logic resource consumptions of flip flops and slice LUTs grow approximately linearly with *M* and *S*, due to parallel fern units and feature extractors; (2) the memory consumption for *N*(*m*, *F_m_*, *c*) grows linearly with *M* and *C*, and exponentially with *S*, as the size along the *F_m_* dimension is 2*^S^* as mentioned earlier; (3) the memory consumption for the logarithmic LUTs in the fern unit array grows linearly with *M*; and (4) the resource consumptions in other blocks roughly remains constant regardless of the hyperparameter values. The hyperparameters *α* and *D* do not affect the resource consumption. These trends are consistent with the observation in Table 1. In any case, the levels of logic resource consumption are relatively small for all the three DVS datasets, and the memory consumption varies significantly depending on the value of *S* used for each dataset. Table 2 also exhibits the estimated levels of power consumption given by the Vivado power analyzer tool. The levels of power consumption of compiled prototype instances for these DVS datasets suggest no significant differences, varying from 690 mW for MNIST-DVS down to 564 mW for Poker-DVS, including an almost constant static power contribution of 230 mW. Typically, the energy efficiency of our prototype for processing the MNIST-DVS can achieve as high as 100 Meps/690 mW = 145 Meps/W or 145 events/μJ.

**Figure 8:**
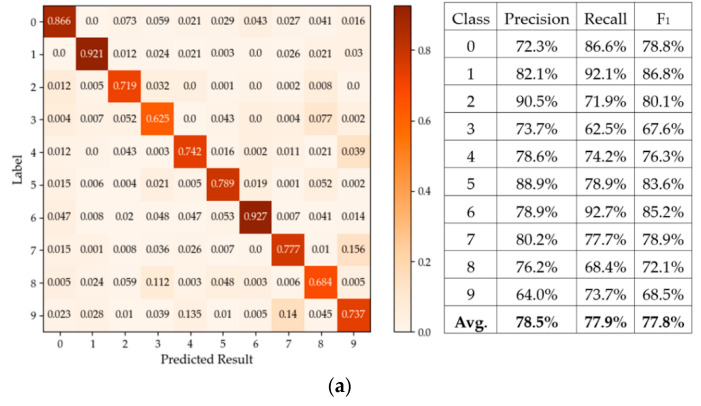

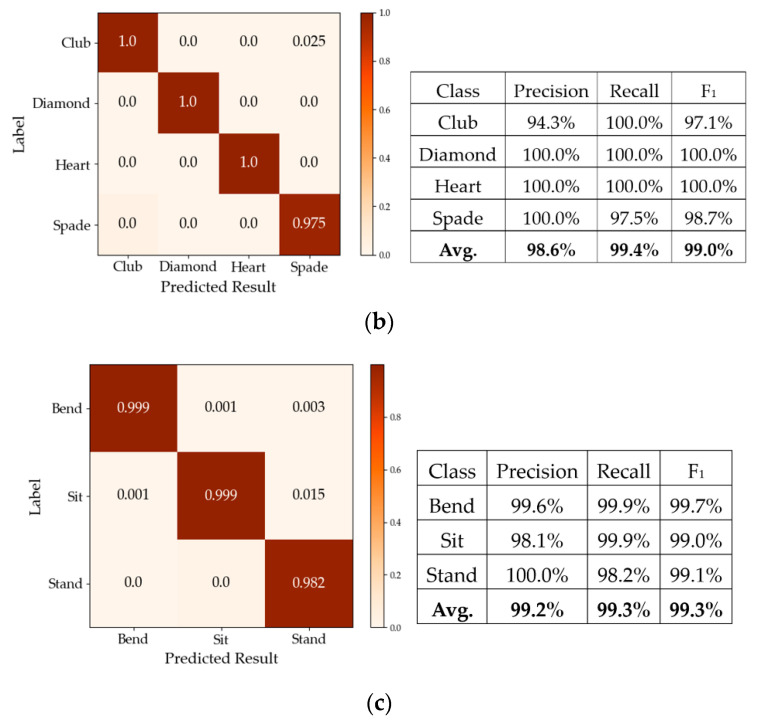
Confusion matrices and precision, recall, F1 metrics for the datasets. (**a**) MINST-DVS, (**b**) Poker-DVS, and (**c**) Posture-DVS.

The prototype evaluation procedure was as follows. First, the DVS data were preprocessed by the PC software. Each DVS dataset contains multiple continuous AER streams [38]. For each dataset, we randomly selected 90% of its AER streams as the training set and the others as the testing set. Each stream was further divided into multiple segments according to the hypermeter *α* for each dataset, as listed in Table 2. Then, these AER segments were downloaded into the on-chip virtual DVS via the Ethernet interface and the ARM IP core. Standard functional Application Programming Interfaces (APIs) were used by PC and ARM software programs to implement the Ethernet communication. Once the virtual DVS held enough AER segments, it started to issue AER events at a rate of 1 event per clock cycle to the VLSI prototype for training or class inference. Finally, if in the class inference phase, the classification results were read by the ARM core and sent back to the PC to be displayed. When the virtual DVS camera was empty, the PC loaded other AER segments onto it and the evaluation procedure repeated. The classification accuracies and the time consumptions on the prototype are given and compared wo other works later in Section 4.2. The classification accuracies on the testing sets of MNIST-DVS, Poker-DVS, and Posture-DVS are 77.9%, 99.4% and 99.3%, respectively. These accuracies were obtained based on 10-fold cross-validations [30]. More details are shown in the confusion matrices in Figure 8. The measured (peak) event throughput on our FPGA prototype with the virtual DVS sensor generating 1 AER event per clock cycle (i.e., 100 M events under the 100 MHz clock cycle) was 100 Meps, and the measured pseudo-simultaneity latencies were 0.21 μs, 0.15 μs and 0.14 μs for the MNIST-DVS, Poker-DVS, and Posture-DVS datasets, respectively. These measurements exactly matched with the theoretical predictions in Section 3.

### 4.2. Comparison and Discussion

To demonstrate the high-speed processing performance of our proposed VLSI hardware system, Table 3 compares our FPGA hardware prototype with recent state-of-the-arts software-based solutions for AER object classification, in terms of classification accuracies, as well as levels of training and classification inference time consumption, on the above three DVS datasets. Those software-based solutions themselves were already compared in [30]. In this work, we further compare them with our proposed hardware system. The studies described in [15,16] adopted spiking convolutional neural networks (SCNN) for AER processing, with one convolutional layer (handcrafted DoG or Gabor filters) and one fully-connected learning layer (trained by the Tempotron rule [13]). In contrast, the works of [28,30] employ lightweight statistical learning methods for AER object classification, and achieve comparable classification accuracies. In particular, the work in [28] uses BoE features and SVM classifier. The work in [30] uses even simpler binary features and Random Ferns, and achieves the highest accuracies. Moreover, the simplest algorithm proposed in [30] has the best potential for hardware acceleration, as implemented in this work. Table 3 shows that the proposed dedicated hardware system achieves speeds hundreds to tens of thousands of times faster than those previous software-based solutions. In particular, the last line of Table 3 exhibits the processing speed gains of our hardware system over its software implementation counterpart of [30]. This demonstrates the excellent algorithm accelerating capability of our dedicate VLSI architecture and circuits. 

Table 4 compares our work with prior state-of-the-art digital hardware VLSI systems that are implemented as small-area application-specific integrated chips (ASICs) or on medium-cost FPGAs for embedded AER visual processing applications. The designs of [22,23,39] were used to run fully-connected SNNs. However, their SNNs are benchmarked on AER spike trains manually converted from MNIST image frames, rather than those directly recorded by DVS cameras. Accordingly, their energy efficiency is measured in terms of the energy consumed in neuronal synaptic operations (Gsops/W, giga synaptic operations per second per Watt) instead of on the input DVS events (Meps/W) in Table 4. In SNNs, processing one input AER event incurs tens to hundreds of synaptic operations. For instance, in a typical 784-200-10 three-layer SNN, any AER event occurring at one of its inputs will trigger at least 200 synaptic operations, as every spiking neuron in the second layer has to add corresponding synaptic weights connected to that input to their membrane potentials. In this case, the 145 Meps/W energy efficiency of our system is equivalent to 145 Meps/W × 200 sop/event = 29 Gsops/W, which is in the same order of magnitude of the energy efficiencies of [23] and [39] in Table 4. Moreover, if our system could be implemented as a custom ASIC chip fabricated using very advanced low-power nanometer technologies, as adopted by [23,39], its energy efficiency can be considerably further improved. In Table 4, the system processing latencies of [22,23,39] are relatively large because one has to first get a complete frame image, then convert it to AER spike trains, and wait for those hardware SNN systems to finish the processing. No pseudo-simultaneity exists between the sensor and the processor, as the spike event cannot be processed on-the-fly with the sensing procedure. Therefore, the system latency is as long as the time consumption for processing all the spike events converted from an image frame. The MNIST digits classification accuracies of [22,23,39] are relatively higher than other hardware implementations in Table 4 and all software implementations in Table 3. Once again, this is due to the fact that the works described in [22,23,39] are benchmarked on static MNIST images. Although these images are converted to AER spike events for processing, the spike timings exactly preserve the original image pixel information. In contrast, the MNIST-DVS benchmark datasets employed by other designs are recorded by DVS cameras with only temporal change information. These DVS events are sparser and more noisy, and less representative of the original MNIST digit images, as illustrated by the pseudo images reconstructed from the DVS AER segments in Figure 7. Correctly Classifying such DVS data is much more challenging than classifying the spike trains manually converted from static images. 

The chips of [24,26,27] were designed to accelerate the convolutional layer processing in SCNNs directly fed by DVS AER events (either from in-situ real DVS cameras or from DVS recorded datasets). The system of [40] is designed to accelerate a dedicate statistical processing method (called Hierarchy of Time-Surfaces, or HOTS) for DVS gesture classification. In Table 4, our hardware systems realized 100 Meps DVS event throughput, about 6~128 times higher than the other DVS event processing systems in [24,26,27,40]. Our system also achieved much higher energy efficiency than them, except for the one in [26] in high-efficiency low-throughput mode. Our system processing latency was as short as 0.21 μs, which is 0.035 μs marginally longer than [24], and negligible to all the other implementations in Table 4. Our system realized near-perfect 99.4% and 99.3% classification accuracies on the Poker-DVS and the Posture-DVS datasets, respectively, while the works of [26,40] exhibit lower accuracies of 97.5% and 93.3% on similar DVS datasets of poker cards and gestures.

However, the main limitation of our high-speed low-cost AER processing system is that it may not cope well with tasks when more than 5 object classes are involved. It can be seen from the above experimental and comparison results that the classification accuracy of our proposed VLSI system degrades when the number of object classes concerned increases. It can arrive at nearly 100% correct rates on the Poker-DVS dataset (containing 4 classes) and the Posture-DVS dataset (containing 3 classes), but only around an 80% correct rate on the MNIST-DVS dataset (containing 10 classes). It is inferior to those SNN-based neuromorphic systems in terms of accuracies when presented with complex and challenging tasks with more object classes, such as classifying the MNIST-DVS dataset. The reason is that our system is dedicated to carrying out the simple and fast Random Ferns algorithm, and hence consumes very few computational resources, trading off accuracies on complex tasks for system throughput and latency improvements, resource reduction, and an energy-efficiency boost. Compared to other neuromorphic systems running complicated SNN algorithms, our proposed VLSI hardware is thus especially appropriate for those applications categorizing only a few object classes or even making binary decisions (i.e., face detection, pedestrian tracking, etc.), with high processing speed, low computing latency, low system cost and high energy efficiency being of critical concerns.

Since real DVS cameras are not available to us at this moment, and we hope to test the peak event throughput of the hardware prototype, we adopted the virtual DVS sensor for evaluation experiments in Section 4.1. However, if our VLSI hardware can be connected to a real DVS retina sensor in future work, a synchronizer must be employed to adapt to the asynchronous AER spike stream outputted from the real DVS sensor. Such a synchronizer has to be run under a 200 MHz clock frequency to maintain the 100 Meps throughput performance of our hardware system. Such a synchronizer can be integrated on the DVS sensor side or on the processing circuit side, or implemented externally on a small FPGA board. Moreover, for more complex AER routing methods that support arbitrary and buffering on simultaneous AER spike outputs, some special interfaces like AER-USB have to be adopted between the AER sensor and processor components [41]. In that case, our processing hardware would not reach its peak event throughput performance, as it has to wait until the arbiter and the interface have completed necessary data preprocessing and rescheduling. 

## 5. Conclusions

In this paper, we propose a high-speed low-cost VLSI hardware system for DVS AER object classification, based on a lightweight statistical algorithm involving simple binary features and the Random Ferns classifier. Our system architecture achieves high event throughput and low processing latency by employing multiple levels of pipelines and parallel processing unit circuits. The memory-centric paradigm of the architecture guarantees the cost-effectiveness of the system. Moreover, our system realizes on-chip online learning for the Random Ferns classifier with very few extra computing circuits. These properties make our system very suitable for many embedded applications requiring high-speed performance, tight cost budgets, and self-adaptiveness via in-situ learning. An FPGA prototype of the proposed VLSI hardware system was implemented on the Xilinx Zynq-7045 chip. Under a 100 MHz clock frequency, the prototype realized 100 Meps event throughput, 145 Meps/W energy efficiency, and 0.21 μs processing latency, and obtained 77.9%, 99.4% and 99.3% classification accuracies on the MNIST-DVS, Poker-DVS, and Posture-DVS datasets, respectively, while consuming a moderate amount of 9.5~96.7% memory resources and a small amount of <11% computational/logic resources on the FPGA chip. Compared to prior state-of-the-art works for AER data processing, our hardware system achieves much higher event throughput, higher energy efficiency, lower processing latency, and higher classification accuracies. 

## Figures and Tables

**Table 1:** Hyperparameter settings for different DVS datasets [30].

Hyperparameter.	*M* (# of Ferns)	*S* (# of Binary Feature)	*α* (# of Segment Event)	*D* (Patch Size Range)	*C*
MNIST-DVS	50	12	300	3, 4 or 5	10
Poker-DVS		8	100		4
Posture-DVS		10	500		3

**Table 2:** Resource and power consumptions of our FPGA prototype for different DVS datasets.

FPGA Platform (Zynq-7045)	Logic Resources			Memory Resources	Power Consumption (mW)
	Flip-Flops ^1^ (437200)	Slice LUTs (218600)	DSP/Multiplier (900)	Block RAM (545)	
MNIST-DVS	46505 (10.6%)	21882 (10.0%)	1 (0.11%)	527 (96.7%)	690
Poker-DVS	31318 (7.2%)	14702 (6.7%)	1 (0.11%)	52 (9.5%)	564
Posture-DVS	38843 (8.9%)	16923 (7.7%)	1 (0.11%)	79 (14.5%)	652

^1^ The Flip-flop resource type is also called Slice Registers in the Vivado implementation tool report.

**Table 3:** Comparison to software-based solutions for AER object classification.

Method	MNIST-DVS			Poker-DVS			Posture-DVS		
	Acc.	T_train_	T_class_	Acc.	T_train_	T_class_	Acc.	T_train_	T_class_
	(%)	(s)	(s)	(%)	(s)	(s)	(%)	(s)	(s)
SCNN [15] (DoG + Tempotron)	62.50	1208	7825	92.53	31	23.34	91.88	1548	118,717
SCNN [16] (Gabor + Tempotron)	75.52	8805	982	91.76	73.12	7.91	95.61	11,794	2984
Statistical Learn. [28] (BoE + SVM)	74.82	31.5	27.4	93.00	0.03	0.02	98.66	45.46	44.64
Statistical Learn. [30] (Random Ferns)	78.08	41.3	5.0	97.2	0.6	0.1	99.59	39.3	5.0
**This work ** **(Random Ferns)**	**77.9**	**0.06** **(×688)**	**0.0066** **(×758)**	**99.4**	**0.00046** **(×1304)**	**0.00006** **(×1667)**	**99.3**	**0.11** **(×357)**	**0.011** **(×456)**

**Table 4:** Comparison to other digital VLSI implementations for AER visual processing.

Design Work	Implementation	Technology	Clock Freq. (MHz)	Power (mW)	DVS Event Throughput (Meps)	Energy Efficiency	Latency (μs)	Algorithm	On-Chip Learning	Benchmark Dataset	Classification Accuracy (%)
Darwin [22]	25 mm^2^ ASIC	180 nm	70	59	N/A	N/A	160,000	SNN	No	MNIST (non-DVS)	93.8
ODIN [23]	0.086 mm^2^ (core) ASIC	28 nm FDSOI	75	0.477	N/A	78.6 Gsops/W ^1^	31,447	SNN	Yes		84.5
[39]	1.72 mm^2^ ASIC	10 nm FinFET	105	9.2	N/A	120.5 Gsops/W	160	SNN	Yes		89
[24]	31.9 mm^2^ ASIC	350 nm	100	200	16.6	83 Meps/W	0.175	SCNN	No	Real DVS camera	N/A
[26]	Spartan-6 FPGA	45 nm	50	7.7	0.05–3	6.4–389 Meps/W	0.5–32	SCNN	No	Poker-DVS	97.5
[27]	Zynq-7100 FPGA	28 nm	100	59	0.779	13.2 Meps/W	9.01	SCNN	No	Poker-DVS	N/A
[40]	Zynq-7100 FPGA	28 nm	100	77	2	26.0 Meps/W	6.7	HOTS	No	NavGestures-sit (DVS)	93.3
**This work**	**Zynq-7045 FPGA**	**28 nm**	**100**	**690**	**100**	**145 Meps/W**	**0.21**	**Random Ferns**	**Yes**	**MNIST-DVS** **Poker-DVS** **Posture-DVS**	**77.9** **99.4** **99.3**

^1^ Gsops = Giga synaptic operations per second.
